# Interictal dysphoric disorder and aggressive behavior in drug-resistant epilepsy: clinical insights

**DOI:** 10.1192/j.eurpsy.2026.11034

**Published:** 2026-07-31

**Authors:** R. Sánchez-González, E. Monteagudo-Gimeno, L. Pintor-Pérez

**Affiliations:** 1 Department of Psychiatry, Institut de Salut Mental, Centre Emili Mira. Hospital del Mar., Barcelona; 2 Department of Psychiatry, Benito Menni Mental Health Care Complex., Sant Boi de Llobregat; 3 Consultation-Liaison Service. Department of Psychiatry., Institut de Neurociències. Hospital Clínic i Provincial de Barcelona. Institut d’Investigacions Biomèdiques August Pi i Sunyer (IDIBAPS) – Universitat de Barcelona., Barcelona, Spain

## Abstract

**Introduction:**

The relationship between aggressive or violent behavior and epilepsy has been a subject of extensive debate. Proposed links between aggression and epilepsy involve multiple factors, including postictal psychoses and rare instances of ictal aggression. However, unlike periictal or ictal violence, interictal violent behavior is generally considered unrelated to epilepsy or specific EEG abnormalities. Furthermore, there is a notable scarcity of research addressing aggressive behavior in the context of Interictal Dysphoric Disorder (IDD), particularly among patients with severe drug-resistant epilepsy (DRE).

**Objectives:**

The primary objective of this study was to examine the potential association between aggressive behavior and interictal psychiatric symptoms, specifically IDD in patients with DRE.

**Methods:**

This cross-sectional, descriptive study included 453 patients with DRE from the Epilepsy Unit at Hospital Clinic, spanning the years 2008 to 2018. Sociodemographic and clinical variables were analyzed. Aggressive behavior was evaluated using the Hostility Subscale of the Symptom Checklist-90, which assesses thoughts, feelings, and actions characteristic of anger, including resentment, irritability, aggression, and rage. Statistical analyses were performed using IBM SPSS Statistics version 23 (Armonk, NY: IBM Corp.) for Microsoft Office 2013. Student’s t-test was employed to compare quantitative variables.

**Results:**

Out of the 453 patients, 132 (29.1%) were diagnosed with Interictal Dysphoric Disorder (IDD), while 321 (70.9%) were not. A statistically significant association was observed between IDD and scores on the Hostility Subscale of the Symptom Checklist-90, with mean scores of 1.22 in the IDD group compared to 0.83 in the non-IDD group (P = 0.000). Table 1 presents the sociodemographic and clinical characteristics of the study sample.

**Table 1.** Table 1. Long description.

Variables	Results
Age (average)	36,9 years
Sex	
Men	196 (43.3%)
Women	257 (56.7%)
Localization of epileptogenic zone	
Temporal	225 (49.6%)
Extratemporal	130 (28.7%)
Not established	98 (21.7%)
Diagnosis of IDD	
Yes	132 (29.1%)
No	321 (70.9%)

**Conclusions:**

Although previous studies have suggested that interictal violent behavior is generally unrelated to epilepsy, our findings demonstrate a significant association between hostile or aggressive behavior and IDD. Originally described by Blumer as a pleomorphic affective disorder in epilepsy, IDD includes paroxysmal irritability among its core symptoms, which may represent the primary factor underlying aggressive behavior and thus explain the observed association. Therefore, we conclude that assessment of violent behavior should be an integral component of the clinical evaluation of patients with IDD.

**Disclosure of Interest:**

None Declared

## Long descriptions

### Table 1. Long description

The table is organized into two columns: Variables and Results.

* Age (average): 36,9 years.

* Sex: Men account for 196 (43.3 percent) and Women account for 257 (56.7 percent).

* Localization of epileptogenic zone: Temporal is 225 (49.6 percent), Extratemporal is 130 (28.7 percent), and Not established is 98 (21.7 percent).

* Diagnosis of I D D: Yes is 132 (29.1 percent) and No is 321 (70.9 percent).

Navigate back to Table 1..

